# Do Individuals with High Climate Anxiety Believe That They Will Die Earlier? First Evidence from Germany

**DOI:** 10.3390/ijerph20065064

**Published:** 2023-03-13

**Authors:** André Hajek, Hans-Helmut König

**Affiliations:** Hamburg Center for Health Economics, Department of Health Economics and Health Services Research, University Medical Center Hamburg-Eppendorf, 20246 Hamburg, Germany

**Keywords:** climate anxiety, climate change, perceived longevity, subjective life expectancy, eco anxiety, natural disaster, flood, longevity, mortality

## Abstract

Objectives: To examine the association between climate anxiety and perceived longevity in the general adult German population (also stratified by age group). Study design: Nationally representative survey. Methods: Data were used of the general adult German population, with n = 3015 individuals (18 to 74 years; data collection: March 2022). Climate anxiety was assessed using the validated Climate Anxiety Scale. It was adjusted for a wide array of covariates in linear-log regression analysis. Results: Even after adjusting for various covariates, there was an association between higher (log) climate anxiety and a lower perceived longevity in the total sample (β = −1.41, *p* < 0.01). Stratified by age group, a significant association was only present among individuals aged 18 to 29 years (β = −3.58, *p* = 0.01), whereas it was not present in the other age groups (i.e., individuals aged 30 to 49 years, individuals aged 50 to 64 years, and individuals aged 65 years and over). Conclusions: This study showed an association between higher climate anxiety and lower perceived longevity, particularly among younger individuals. More clearly, younger individuals with a higher climate anxiety think they will die earlier. This is the first study on this topic and could serve as a foundation for upcoming research. For example, longitudinal studies are needed to confirm our findings.

## 1. Introduction

It is widely accepted that currently a change in the world’s climate is taking place. It is also known that the ongoing climate change is a serious threat to well-being and even mankind’s survival [1]. Therefore, it is very plausible that individuals can develop fears related to climate change. Such climate anxiety was defined by Pikhala as “anxiety which is significantly related to anthropogenic climate change” [2].

Recent studies have examined some potential consequences of climate anxiety. For example, it is associated with higher levels of both loneliness and perceived social isolation [3]. Moreover, higher climate anxiety can contribute to poor mental health [4,5,6,7]. However, to date, there is a complete lack of studies examining the association between climate anxiety and perceived longevity. Our aim was thus to examine this association in the general adult German population (also stratified by age group).

Why is it important to examine such a link? Knowledge about an association between climate anxiety and perceived longevity may be important to gain a better understanding of individuals with higher levels of climate anxiety. Such knowledge could be of importance to policymakers, clinicians, mental health researchers, geriatricians and psychiatrists. Moreover, a lower perceived longevity (e.g., related to a higher climate anxiety) may become a self-fulfilling prophecy [8,9,10]. This means that such individuals scoring high in climate anxiety may develop poor lifestyle habits over time (e.g., higher alcohol intake, a higher likelihood of smoking, or a sedentary lifestyle). Potential reasons for such bad lifestyle habits may be: (i) hopelessness, (ii) anger and incomprehension about those who, in their view, have caused or continue to cause climate change, as well as (iii) feelings of powerlessness in the face of the consequences of climate change. In the long term, such bad lifestyle habits could contribute to morbidity and a reduced actual longevity [11,12]. Thus, such knowledge may be of great importance for successful aging in general.

With regard to potential mechanisms underlying a potential association between climate anxiety and perceived longevity: Factors related to higher climate anxiety such as unsatisfied social needs [11,13] or poor mental health [14] may affect perceived longevity. Furthermore, individuals with a high climate anxiety may particularly fear the individual negative consequences of deadly natural disasters (e.g., floods) or global warming on health or even survival. This could also explain such a link. Moreover, we assume that the link between climate anxiety and perceived longevity is particularly present among younger adults. The reason is that younger individuals scoring high in climate anxiety may fear the long-term consequences of climate change (e.g., natural disasters such as floods) for their own survival, whereas it may be the case that older adults scoring high in climate anxiety do not fear the long-term consequences of climate change for their own health, as they are unlikely to survive for many decades. It may be more likely that most older adults rather fear more immediate health threats such as frailty, falls, immobility or cognitive impairment [15].

## 2. Materials and Methods

### 2.1. Sample

We made use of cross-sectional data from 3091 individuals living in Germany, whose age ranged from 18 to 74 years. This means that individuals younger than 18 years and those aged 75 years and over were excluded. The data was gathered between 15 March and 21 March 2022.

The well-known market research company Bilendi & respondi (an online sampling service with ISO 26362 certification) invited participants. They were chosen from a quota-based online sample to assure that the respondents’ age, gender, and distribution by federal state accurately represented the demographics of the adult German population as a whole. Overall, 11,900 people were invited to attend. Because the sample was obtained online, a potential selection bias could not be calculated. This should be acknowledged as a potential limitation.

Each individual provided their informed consent. The Psychological Ethics Committee of the of the University Medical Center Hamburg-Eppendorf approved this study (LPEK-0412).

### 2.2. Dependent Variables

The expected longevity was quantified in accordance with former research [11,13]. Individuals were asked “What age do you think you will live to?” [_ _ _ years]. Against the backdrop of the present highest confirmed ages (about 120 years), rather implausible values (higher than 120 years; for example, an expected longevity of 999 years) or invalid values (e.g., text answers translating to “old”; when the perceived longevity is lower than the chronological age) were removed. This resulted in 76 cases being eliminated in total.

### 2.3. Independent Variable of Interest

The Climate Anxiety Scale (CAS) developed by Clayton and Karazsia [16] was used to measure climate anxiety. Consisting of 13 items (ranging from 1 = strongly disagree to 7 = strongly agree/applies totally), this instrument comprises two factors (functional impairment and cognitive-emotional impairment [16]). The validated [17] German version—which also used in recent research [18]—was used in this current study. The wording was as follows: “Thinking about climate change makes it difficult for me to concentrate.”; “Thinking about climate change makes it difficult for me to sleep.”; “I have nightmares about climate change.”; “I find myself crying because of climate change.”; “I think, ‘why can’t I handle climate change better?’”; “I go away by myself and think about why I feel this way about climate change.”; “I write down my thoughts about climate change and analyze them.”; “I think, ‘why do I react to climate change this way?’”; “My concerns about climate change make it hard for me to have fun with my family or friends.”; “I have problems balancing my concerns about sustainability with the needs of my family.”; “My concerns about climate change interfere with my ability to get work or school assignments done.”; “My concerns about climate change undermine my ability to work to my potential.”; “My friends say I think about climate change too much.”

The items’ average was used to create a final climate anxiety score (from 1 to 7, whereby higher values correspond to a higher level of climate anxiety). In our study, Cronbach’s alpha was 0.95 (McDonald’s omega: 0.95).

### 2.4. Covariates

Based on prior research [11,13], a wide array of covariates was used in regression analysis. More precisely, several sociodemographic, lifestyle-related and health-related covariates were included (see also: [3]).

Regarding sociodemographic factors, age (in years), sex (men; women; diverse), family situation (married, cohabiting with spouse; married, not cohabiting with spouse; divorced; widowed; single), employment status (employed full-time; retired; other), and highest school education (upper secondary school; qualification for applied upper secondary school; polytechnic secondary school; intermediate secondary school; currently in school training/education; without school-leaving qualification/lower secondary school) were included in the regression analysis.

Regarding lifestyle-related covariates, the following factors were included in the regression analysis: smoking behavior (never smoker; no, not anymore; yes, sometimes; yes, daily), alcohol intake (daily; several times a week; once a week; 1–3 times a month; less often; never), frequency of sports activities (regularly, more than 4 h a week; regularly, 2–4 h a week; regularly, 1–2 h a week; less than one hour a week; no sports activity).

With regard to health-related covariates, we included self-rated health (from 1 = very bad to 5 = very good), having at least one chronic condition (no; yes), vaccination against COVID-19 (no; yes), and probable anxiety. The Generalized Anxiety Disorder-7 Assessment (GAD-7 [19]) was used to assess probable anxiety. Based on seven items, a sum score was developed. This sum score ranges from 0 to 21 (higher values indicate more anxiety symptoms). For detection of generalized anxiety disorder (using a GAD-7 score at ≥10), the specificity was 0.82 and the sensitivity was 0.89. Based on the given recommendations [19], a cut-off score of ≥10 was used for probable anxiety. In this study, Cronbach’s alpha was 0.91 (McDonald’s omega: 0.91).

### 2.5. Statistical Analysis

First, the sample characteristics are displayed for the entire analytical sample as well as the different age groups. Effect sizes (Pearson’s r)—were additionally calculated for the association between climate anxiety and perceived longevity (for the total sample and also for the different age groups). Thereafter, multiple linear-log regressions were performed to study the association between climate anxiety and perceived longevity—accounting for a wide array of covariates among the total sample and additionally stratified by age group. Using linear-log regressions means that the natural logarithm of climate anxiety was used as a key independent variable, because of the right-skewed distribution of climate anxiety. In the sensitivity analysis, we used a full-information maximum likelihood approach to tackle missing data [20]. In an exploratory fashion, we examined whether education (trichotomized: polytechnic secondary school or higher; intermediate secondary school; lower than intermediate secondary school) moderates the association between (log) climate anxiety and perceived longevity in the total sample.

We used a recently developed tool to determine McDonald’s omega [21] with the significance level set at *p* < 0.05. For statistical analyses, Stata 16.1 (Stata Corp., College Station, TX, USA) was used in this current study.

## 3. Results

### 3.1. Sample Characteristics and Pearson Correlations

The sample characteristics for the analytical sample are shown (total sample and for the different age groups). In the analytical sample, n equaled 3015 individuals. In the total sample, average age was 46.5 years (SD: 15.3 years, 18 to 74 years), with 49.9% of the individuals being female. In sum, 59.2% were married, cohabiting with spouse. Additionally, 44.1% of the individuals were full-time employed. Furthermore, 40.0% of the individuals had an upper secondary school education, 46.1% had at least one chronic condition and 88.5% were vaccinated against COVID-19.

Moreover, average climate anxiety was 2.0 (SD: 1.2) and average perceived longevity was 80.1 years (SD: 12.7 years). Significant differences between the age groups were present for all variables. Please see Table 1 for further details.

Pearson correlations between climate anxiety and perceived longevity were as follows: r = −0.13, *p* < 0.001 (among the total sample); r = −0.21, *p* < 0.001 (among individuals aged 18 to 29 years); r = −0.09, *p* < 0.01 (among individuals aged 30 to 49 years); r = −0.06, *p* = 0.05 (among individuals aged 50 to 64 years); and r = −0.003, *p* = 0.95 (among individuals aged 65 to 74 years).

### 3.2. Regression Analysis

The results of multiple linear-log regressions are shown in Table 2 (total sample as well as stratified by age group). Adjusted for age, sex, family status, employment status, educational level, smoking status, frequency of sports activities, alcohol consumption, self-rated health, chronic conditions, vaccination status, and probable anxiety in regression analysis, R^2^ varied from 0.15 to 0.20. The presence of multicollinearity was checked. However, the highest variance inflation factors (VIFs) were less than four and the mean VIF was 1.71, indicating that multicollinearity is not a concern.

Regressions revealed that logarithmized higher climate anxiety was associated with lower perceived longevity among the total sample (β = −1.41, *p* < 0.01) and among individuals aged 18 to 29 years (β = −3.58, *p* = 0.01), whereas it was not significantly associated with perceived longevity in the other age groups (i.e., among individuals aged 30 to 49 years, 50 to 64 years, and 65 to 74 years). Further details are shown in Table 2.

In the sensitivity analysis, a FIML approach was used to tackle missing data. In this analysis, the association between logarithmized climate anxiety and perceived longevity remained virtually the same in terms of significance and effect size (e.g., β = −1.41, *p* < 0.01 among the total sample; β = −3.58, *p* = 0.01 among individuals aged 18 to 29 years).

Moreover, we also examined whether education moderates the association between (log) climate anxiety and perceived longevity in the total sample. However, the interaction terms did not achieve statistical significance (low education x log climate anxiety, β = 0.05, *p* = 0.98; medium education x log climate anxiety, β = 0.93, *p* = 0.36).

## 4. Discussion

This study examined the association between climate anxiety and perceived longevity based on data from the general German adult population. Regressions showed an association between higher climate anxiety and a lower perceived longevity among the total sample. Stratified by age group, a significant association was only present among individuals aged 18 to 29 years, whereas it was not present in the other age groups (i.e., individuals aged 30 to 49 years, 50 to 64 years, and 65 years and over).

The fact that a significant association between higher climate anxiety and lower perceived longevity was only present among younger individuals is in accordance with our initial expectations. Prior research also showed that there was a link between higher climate anxiety and higher loneliness/social isolation particularly among younger individuals, whereas no significant association was found among older adults [3]. Such feelings of not belonging to society or loneliness may contribute to a lower perceived longevity—as two recent studies have demonstrated [11,13]. Furthermore, a high climate anxiety can also lead to lower mental health [4,5,6,7]—which in turn can lead to a lower perceived longevity [14].

Another more obvious possible explanation is that younger individuals who exhibit high levels of climate anxiety may particularly fear the short-, medium- and long-term consequences of climate change for their own lives. For example, they could fear deadly natural disasters such as the floods taking place in Europe in the summer of 2021 where almost 200 individuals died in Germany. Moreover, younger adults may be more likely to believe that they live in a time when they will experience the full force of the potential drastic effects of climate change in the future. However, future research is required to substantiate our assumptions.

Some of the strengths and shortcomings of the current study are worth noting. Our study sheds first light on the link between climate anxiety and perceived longevity. It was adjusted for a wide array of covariates. Moreover, a FIML [20] approach was used to address missing values. Furthermore, a valid tool was used to quantify climate anxiety. Data were used from a quota-based sample of the general adult population in Germany (state, age bracket and sex). However, it should be acknowledged that the questionnaire was only available in the German language. Thus, individuals with poor German language skills are most likely be underrepresented. Moreover, access to the internet was a prerequisite for participation. Consequently, certain groups may have a lower likelihood of participation. Additionally, our study is restricted by its cross-sectional design.

## 5. Conclusions

Our current study revealed an association between higher climate anxiety and lower perceived longevity, particularly among younger individuals. More clearly, younger individuals with a higher climate anxiety think that they will die earlier. This is the first study on this topic and could serve as a fundament for upcoming studies in this new research area, for example, longitudinal studies are needed to confirm our findings. Moreover, studies from other countries as well as other age groups (such as the elderly, as well as children and teenagers) are needed. For example, it has been shown that climate anxiety and its consequences can differ by country [22,23,24].

Additionally, it may be worth examining the association between climate anxiety and perceived longevity immediately after a natural disaster (that has taken place nearby) or among individuals directly affected by natural disasters such as floods.

## Figures and Tables

**Table 1:** Sample characteristics for the analytical sample (total sample and for the different age groups).

Variables	Individuals Aged 18 to 29 Years	Individuals Aged 30 to 49 Years	Individuals Aged 50 to 64 Years	Individuals Aged 65 to 74 Years	Total Sample	*p*-Value
	Mean (SD) /n (%)	Mean (SD) /n (%)	Mean (SD) /n (%)	Mean (SD) /n (%)	Mean (SD) /n (%)	
Gender						<0.001
Male	118 (20.8)	482 (46.3)	581 (59.7)	324 (74.7)	1505 (49.9)	
Female	447 (79.0)	556 (53.4)	392 (40.2)	110 (25.3)	1505 (49.9)	
Diverse	1 (0.2)	3 (0.3)	1 (0.1)	0 (0.0)	5 (0.2)	
Marital status						<0.001
Single/Divorced/Widowed/Married, not cohabiting with spouse	301 (53.2)	365 (35.1)	405 (41.6)	159 (36.6)	1230 (40.8)	
Married, cohabiting with spouse	265 (46.8)	676 (64.9)	569 (58.4)	275 (63.4)	1785 (59.2)	
Education						<0.001
Upper secondary school	328 (58.0)	469 (45.1)	278 (28.5)	131 (30.2)	1206 (40.0)	
Qualification for applied upper secondary school	73 (12.9)	138 (13.3)	93 (9.5)	44 (10.1)	348 (11.5)	
Polytechnic secondary school	5 (0.9)	28 (2.7)	110 (11.3)	45 (10.4)	188 (6.2)	
Intermediate secondary school	120 (21.2)	334 (32.1)	352 (36.1)	125 (28.8)	931 (30.9)	
Lower secondary school	27 (4.8)	70 (6.7)	136 (14.0)	88 (20.3)	321 (10.6)	
Currently in school training/education	13 (2.3)	1 (0.1)	1 (0.1)	1 (0.2)	16 (0.5)	
Without school-leaving qualification	0 (0.0)	1 (0.1)	4 (0.4)	0 (0.0)	5 (0.2)	
Employment status						<0.001
Full-time employed	207 (36.6)	620 (59.6)	480 (49.3)	23 (5.3)	1330 (44.1)	
Retired	1 (0.2)	38 (3.7)	209 (21.5)	382 (88.0)	630 (20.9)	
Other	358 (63.3)	383 (36.8)	285 (29.3)	29 (6.7)	1055 (35.0)	
Smoking status						<0.001
Yes, daily	80 (14.1)	254 (24.4)	295 (30.3)	81 (18.7)	710 (23.5)	
Yes, sometimes	58 (10.2)	83 (8.0)	66 (6.8)	23 (5.3)	230 (7.6)	
No, not anymore	117 (20.7)	276 (26.5)	316 (32.4)	211 (48.6)	920 (30.5)	
Never smoker	311 (54.9)	428 (41.1)	297 (30.5)	119 (27.4)	1155 (38.3)	
Alcohol intake						<0.001
Daily	15 (2.7)	35 (3.4)	81 (8.3)	63 (14.5)	194 (6.4)	
Several times a week	56 (9.9)	165 (15.9)	211 (21.7)	97 (22.4)	529 (17.5)	
Once a week	93 (16.4)	179 (17.2)	125 (12.8)	55 (12.7)	452 (15.0)	
1–3 times a month	136 (24.0)	189 (18.2)	149 (15.3)	61 (14.1)	535 (17.7)	
Less often	149 (26.3)	284 (27.3)	223 (22.9)	82 (18.9)	738 (24.5)	
Never	117 (20.7)	189 (18.2)	185 (19.0)	76 (17.5)	567 (18.8)	
Sports activities						<0.001
No sports activity	93 (16.4)	222 (21.3)	349 (35.8)	160 (36.9)	824 (27.3)	
Less than one hour a week	112 (19.8)	206 (19.8)	162 (16.6)	82 (18.9)	562 (18.6)	
Regularly, 1–2 h a week	153 (27.0)	291 (28.0)	214 (22.0)	85 (19.6)	743 (24.6)	
Regularly, 2–4 h a week	114 (20.1)	181 (17.4)	122 (12.5)	64 (14.7)	481 (16.0)	
Regularly, more than 4 h a week	94 (16.6)	141 (13.5)	127 (13.0)	43 (9.9)	405 (13.4)	
Vaccinated against COVID-19						<0.01
Not vaccinated	63 (11.1)	145 (13.9)	101 (10.4)	37 (8.5)	346 (11.5)	
Vaccinated	503 (88.9)	896 (86.1)	873 (89.6)	397 (91.5)	2669 (88.5)	
Chronic diseases						<0.001
Absence of at least one chronic disease	406 (71.7)	679 (65.2)	395 (40.6)	144 (33.2)	1624 (53.9)	
Presence of at least one chronic disease	160 (28.3)	362 (34.8)	579 (59.4)	290 (66.8)	1391 (46.1)	
Self-rated health	3.9 (0.8)	3.8 (0.8)	3.3 (0.9)	3.3 (0.9)	3.6 (0.9)	<0.001
Probable anxiety						<0.001
Absence of probable anxiety	410 (72.4)	880 (84.5)	842 (86.4)	404 (93.1)	2536 (84.1)	
Presence of probable anxiety	156 (27.6)	161 (15.5)	132 (13.6)	30 (6.9)	479 (15.9)	
Climate anxiety	2.4 (1.3)	2.1 (1.3)	1.8 (1.0)	1.8 (0.9)	2.0 (1.2)	<0.001
Perceived longevity (in years)	78.0 (17.0)	79.3 (13.6)	80.6 (10.2)	83.4 (7.4)	80.1 (12.7)	<0.001

Notes: One-way ANOVAs or Chi-squared tests were conducted, as appropriate (*p*-values). Self-rated health ranged from 1 (very bad) to 5 (very good). The climate anxiety score ranged from 1 to 7, whereby higher values correspond to a higher level of climate anxiety.

**Table 2:** Climate anxiety and perceived longevity. Results of linear-log regressions.

Independent Variables	Perceived Longevity—Total Sample	Perceived Longevity—among Individuals Aged 18 to 29 Years	Perceived Longevity—among Individuals Aged 30 to 49 Years	Perceived Longevity—among Individuals Aged 50 to 64 Years	Perceived Longevity—among Individuals Aged 65 to 74 Years
(Log) climate anxiety	−1.41 ** (0.50)	−3.58 * (1.44)	−0.82 (0.82)	−0.85 (0.71)	0.01 (0.79)
Potential confounders	√	√	√	√	√
R^2^	3015	566	1041	974	434
Observations	0.15	0.19	0.17	0.18	0.20

Unstandardized beta-coefficients are reported; robust standard errors in parentheses; ** *p* < 0.01, * *p* < 0.05; potential confounders cover age, sex, family status, employment status, educational level, smoking status, alcohol intake, frequency of sports activities, self-rated health, chronic conditions, vaccination status, and probable anxiety.

## Data Availability

The datasets generated and/or analyzed during the current study are not publicly available due to legal restrictions but are available from the corresponding author on reasonable request.
